# Supplementary material for: A retrospective observational study on maternal and neonatal outcomes of COVID-19: Does the mild SARS-CoV-2 infection affect the outcome?
Source: PeerJ. 2023 Dec 14;11:e16651. doi: 10.7717/peerj.16651 (PMC10725666; doi:10.7717/peerj.16651)
Supplement: Supplemental Information 1 [file peerj-11-16651-s001.docx]

Table S1. The NIH criteria for clinical classification of COVID-19 patients

| COVID-19 clinical classification | | Description |
| --- | --- | --- |
| Asymptomatic or presymptomatic | Individuals who test positive for SARS-CoV-2 using a virologic test (i.e., a nucleic acid amplification test or an antigen test) but have no symptoms consistent with COVID-19. | |
| Mild | Individuals who have any of the various signs and symptoms of COVID-19 (e.g., fever, cough, sore throat, malaise, headache, muscle pain, nausea, vomiting, diarrhea, loss of taste and smell) but do not have shortness of breath, dyspnea, or abnormal chest imaging. | |
| Moderate | Individuals who show evidence of lower respiratory disease during clinical assessment or imaging and who have an oxygen saturation measured by pulse oximetry (SpO2) ≥94% on room air at sea level. | |
| Severe | Individuals who have SpO2 <94% on room air at sea level, a ratio of arterial partial pressure of oxygen to fraction of inspired oxygen (PaO2/FiO2) <300 mm Hg, a respiratory rate >30 breaths/min, or lung infiltrates >50%. | |
| Critical | Individuals who have respiratory failure, septic shock, and/or multiple organ dysfunction. | |
